# AI designed, mutation resistant broad neutralizing antibodies against multiple SARS-CoV-2 strains

**DOI:** 10.1038/s41598-025-98979-w

**Published:** 2025-05-03

**Authors:** Yue Kang, Kevin Jin, Lurong Pan

**Affiliations:** Ainnocence Inc., Suite B PMB 1147, Mountain View, CA 94040 USA

**Keywords:** Biochemistry, Biophysics, Computational biology and bioinformatics, Drug discovery

## Abstract

In this study, we developed a digital twin for SARS-CoV-2 by integrating diverse data and metadata with multiple data types and processing strategies, including machine learning, natural language processing, protein structural modeling, and protein sequence language modeling. This approach enabled us to computationally design neutralizing antibodies against over 1300 historical strains of SARS-CoV-2, encompassing 64 mutations in the receptor binding domain (RBD) region. 70 AI-designed antibodies were experimentally validated through binding assay and real viral neutralization assays against various strains, including later Omicron strains do not present in the initial design database. 14% of these antibodies exhibited strong reactivity against the RBD of multiple strains, achieving triple cross-binding hit rates using ELISA assay. 10 antibodies neutralized the cytopathic effects (CPE) of the Delta strain at IC50 values of < 10 µg/ml, and one antibody neutralized the CPE of Omicron. These findings demonstrate the potential of our approach to influence future therapeutic design for existing virus strains and predict hidden patterns in viral evolution that AI can leverage to develop emerging antiviral treatments.

## Introduction

The evolution of new strains of SARS-CoV-2 has rendered many previously approved antibody therapeutics ineffective, especially those that target the spike protein of the virus. Among the hundreds of various mutations in its genome. Those within the ACE2 receptor binding domain (RBD) of the spike protein have been the major focus for researchers, as these mutations drastically affect the binding strength of the spike protein with the ACE2 receptor. For instance, the L452R substitution found in the B.1.427 and B.1.429 lineages significantly reduce virus’s susceptibility to bamlanivimab^1^, as well as modestly decreasing its susceptibility to the combination of bamlanivimab and etesevimab^1–3^.

In the present study, we used artificial intelligence (AI) to generate more than ${10}^{9}$ antibody mutations in silico and then virtually screen antibody sequences for candidates that can bind broadly and with high affinities to known spike protein RBD variants.

Graph neural networks (GNNs)^4^ are neural network architectures designed specifically to cope with graph data. Nodes in the graph are designed to learn an embedding that contains information about their associated neighbors. The embeddings can function as characteristic features for node labeling, edge prediction, and graph representation with proper readout and pooling methods^5–8^. The intrinsic design of GNN makes it well-suited to study molecular and biological interactions and other chemical and physical properties. We therefore seek to describe antibody-antigen interactions in a graph-based manner.

Language-based networks can also model proteins based on the assumption that the primary protein structure is analogous to natural language sequences^9–14^. Hidden dependencies and interactions between amino acids may be trainable by the temporal dynamic inherently designed in the basic recurrent neural networks, such as long short-term memory networks (LSTM) and transformer neural networks.

We explored several modeling strategies for GNN and natural language processing architectures, in which protein sequences are described using graph-based and language-based representations, respectively.

Overall, this study describes an AI-based approach using deep learning which can capture information for antibody-antigen binding using the protein sequences without any additional data. This model can predict the mutational impact of the protein-protein interaction for rapidly evolving targets such as the different strains of SARS-CoV-2. Our study describes using a deep learning model to computationally design effective and broad-spectrum mutations against various strains of the virus’ spike protein, and subsequent wet-lab experimentation confirms the findings. Because of the efficient nature of this Al-driven antibody discovery approach, it may be used for the rapid discovery of therapeutics in future pandemics. This approach also opens new doors for the design of conventional protein drug discovery that can bind to multiple antigens either broadly or with optimized selectivity.

## Methods

### In silico antibody affinity maturation modeling via AI

#### Training and testing datasets

We have developed antibody affinity maturation Al models based on deep neural networks. We examined the following publicly available curated datasets for model development: SKEMPI database^15^ Antibody-Bind (AB-Bind) database^16^, Observed Antibody Space^17^, and UniProt^18^. The resulting model’s performance was validated on SKEMPI and AB-Bind, both of which curate a protein-protein complex with relevant PDB structures, along with single- and multiple-site mutations. In this data curation process, only the sequence data from this protein-protein complex is used for model training. AB-Bind represents the binding affinities for mutated variants by the change in free energy) (ΔΔG) of binding in $\text{kcal}/\text{mol}$, whereas SKEMPI provides experimental dissociation constant (Kd) values for the affinities of both wildtype complexes and mutants.

#### Deep learning models

**Fig. 1:**
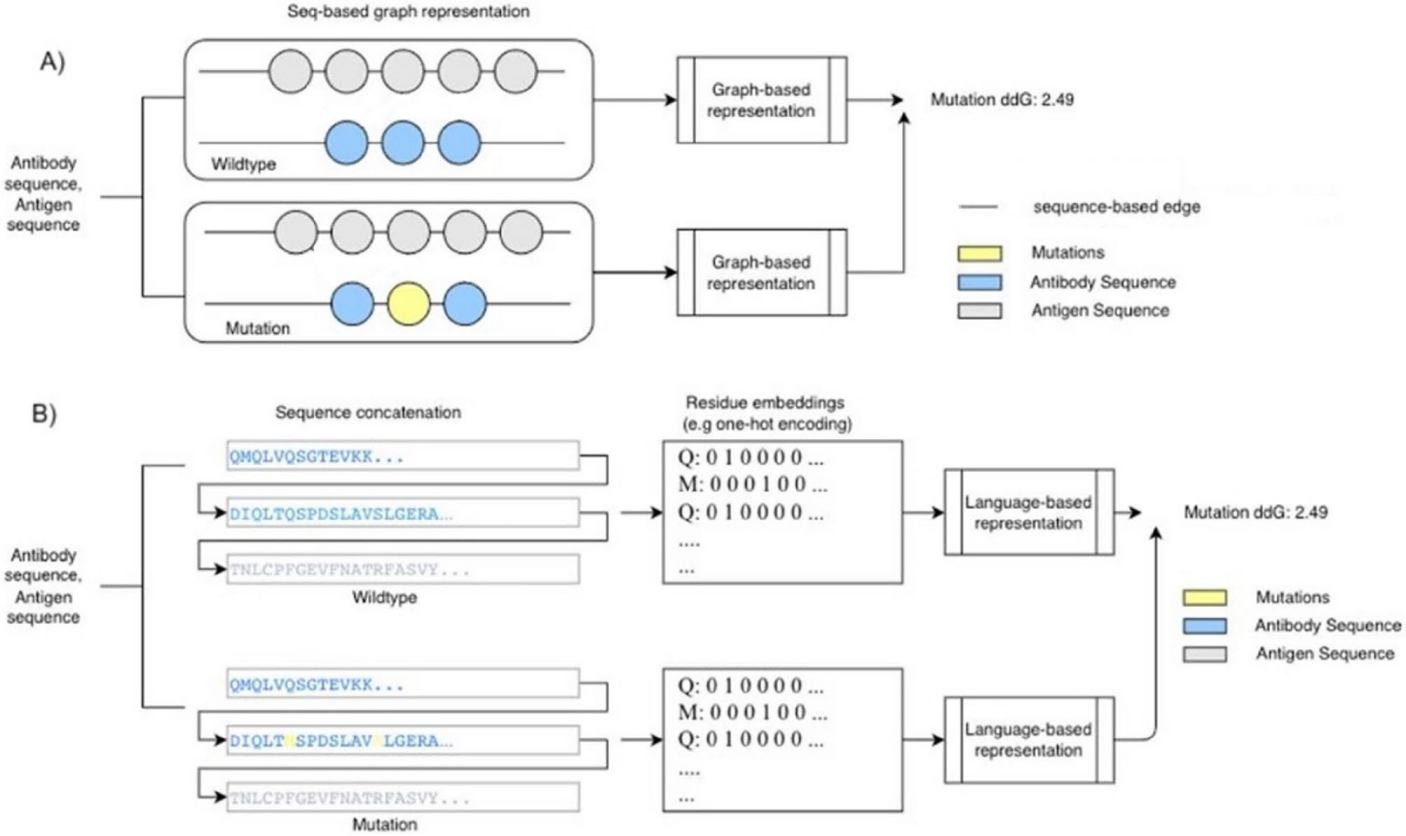
Design of the two modeling approaches developed to support sequence-based antibody affinity design. (**A**) Graph neural network representation for affinity training (**B**) language-based (BiLSTM or transformer) neural network representation for affinity training.

This study aims to examine whether antibody-antigen complex affinities can be predicted using only primary sequence inputs. Therefore, to reduce computing cost and extend the model application to proteins (the structures of which have not been solved), we explored a model that requires only a protein’s amino acid sequence as input to predict binding affinity. Figure 1 illustrates two examples of modeling strategies.

In our study, two different approaches were designed and implemented to predict binding, the graph representation and language-based representation. The graph-based representation utilizes a neural network based on a heterogeneous aggregation graph neural network architecture^19^. This would allow the GNN to form clear representations of the graph by aggregating multiple readout layers. GNNs are used for this sequence-based approach since they propagate nodal information to neighbor nodes^19^ allowing the local protein sequence features to be represented by the model. For the language-based representation, two different architectures were considered, a Bidirectional LSTM (BiLSTM) model and a Transformer model. The BiLSTM was chosen to represent the protein sequence since the primary structure of the protein does not necessarily correlate to the 3D shape of the protein; therefore, the forward and backward context are both important when modeling the protein sequence. The transformer model^20^ uses a pretrained model’s embeddings (a vector representation) from a large amount of data using databases such as UniRef50^21^.

Classification tasks predict binding affinity variations between mutated antibodies and given antigens. We consider mutations that result in either increased (i.e. $\Delta \Delta {\text{G}} < 0$) or decreased (i.e. $\Delta \Delta {\text{G}} > 0$) binding affinities as positive or negative samples, respectively. Our model seeks to extract latent properties of amino acids that can be best used to optimally delineate between “strengthened” and “weakened” binders.

For the GNN model, a model based on heterogeneous aggregations between the GNN layers was used as the primary architecture. Graph data normally contain some information as nodes and connections between nodes as edges. In this case, each amino acid residue is encoded as a numerical value representing one node, and the edges are the connections between the nodes. This GNN was trained using 5 graph convolution layers and pyramid feature stacking was used from each of the readout layers. Readouts of all layers are summed together, and dense connections are added amongst all blocks. Additionally, this model was trained using a maximum of 500 epochs following the “leave-5-out” (L5O) approach for each fold. Each epoch was trained using the SGD optimizer with a learning rate of 0.02 and weight decay of 0.0001.

In the language model, sequence data was used differently for the protein sequence representations. First, in the BiLSTM model, the individual amino acid residues were converted into numerical one-hot encoding representations. This representation was encoded in the same order as the protein sequence with the size of $20 * N$ where N represents the maximum length of the protein sequence. Sequences shorter than this length were padded with zeros. This representation was input into the 3 LSTM layers and subsequent dense layers. Again, this model was trained for a maximum of 500 epochs with the “leave-5-out” (L5O) approach for each fold. Each epoch was trained using the SGD optimizer with a learning rate of 0.02. Next, using the ProtBERT and ESM2 model, embeddings were encoded from the default pretrained model^20^ parameters. This was then passed directly into dense layers and trained using the SGD optimizer with a learning rate of 0.02 for 500 epochs.

#### Model evaluation

We evaluated the model’s accuracy and scalability using out-of-distribution cross-validation based on protein family distinctions. Our validation strategy employed a ‘leave-5-out’ (L5O) approach, where five protein families and their associated complexes (both wildtype and mutations) were randomly withheld as the validation set while the remaining data served as the training set. This design ensured the model encountered completely novel sequence patterns during testing.

From our curated dataset containing 60 unique protein families, each validation fold reserved 5 randomly selected protein families, guaranteeing that validation proteins remained entirely out-of-distribution from the training data (Fig. S1). To confirm the unbiased nature of this sampling method, we conducted a comprehensive similarity analysis across all 60 protein families using pairwise global alignment percent identity scores. The maximum observed similarity between any two protein families was only 74%, with most scoring significantly lower. Given that the average protein sequence in our dataset exceeds 500 residues, even the most similar families differed by approximately 130 amino acid residues, establishing true out-of-distribution conditions between training and testing sets. The resulting trained models were subsequently refined and integrated into SentinusAI®, our purpose-built, structure-free large molecule platform.

In practice, SentinusAI®’s in-silico affinity maturation process emulates the natural immune system by systematically searching for high-affinity binders to specified targets/antigens within a comprehensive virtual library of mutants. This computational approach enables exploration of a somatic mutation space vastly exceeding the capacity of any wet lab methodology. Through optimized engineering and distributed computation strategies, we have achieved computational efficiency that is 10,000-fold faster than traditional methods, allowing rapid identification of optimal antibody candidates that would otherwise require prohibitively extensive experimental screening.

### Computational workflow for identifying COVID-19 neutralizing antibodies

#### Data collection

We retrieved information on over 1300 different historical SARS-CoV-2 strains (including wildtype (B.1) and Delta) from the GISAID^22–24^ database as of August 26, 2021. Three wildtype antibodies, CR3022, Casirivimab (Regen 10,933), and Imdevimab (Regen 10987) were chosen as templates for in silico cross-binding antibody design. The CR3022 antibody^25^ is a monoclonal antibody specific to SARS-CoV-1 and was obtained from human convalescent plasma in a patient that had recovered from severe acute respiratory syndrome (SARS-CoV-1), a virus closely related to the novel coronavirus that causes COVID-19. CR3022 cross-reacts with the novel coronavirus, although its binding affinity is not enough to neutralize the virus and stop it from infecting cells^25–28^. Both Casirivimab and Imdevimab^29^, are monoclonal antibody cocktails developed by Regeneron; their potencies against wildtype and Delta strains have been validated.

#### In silico mutant library generation

**Fig. 2:**
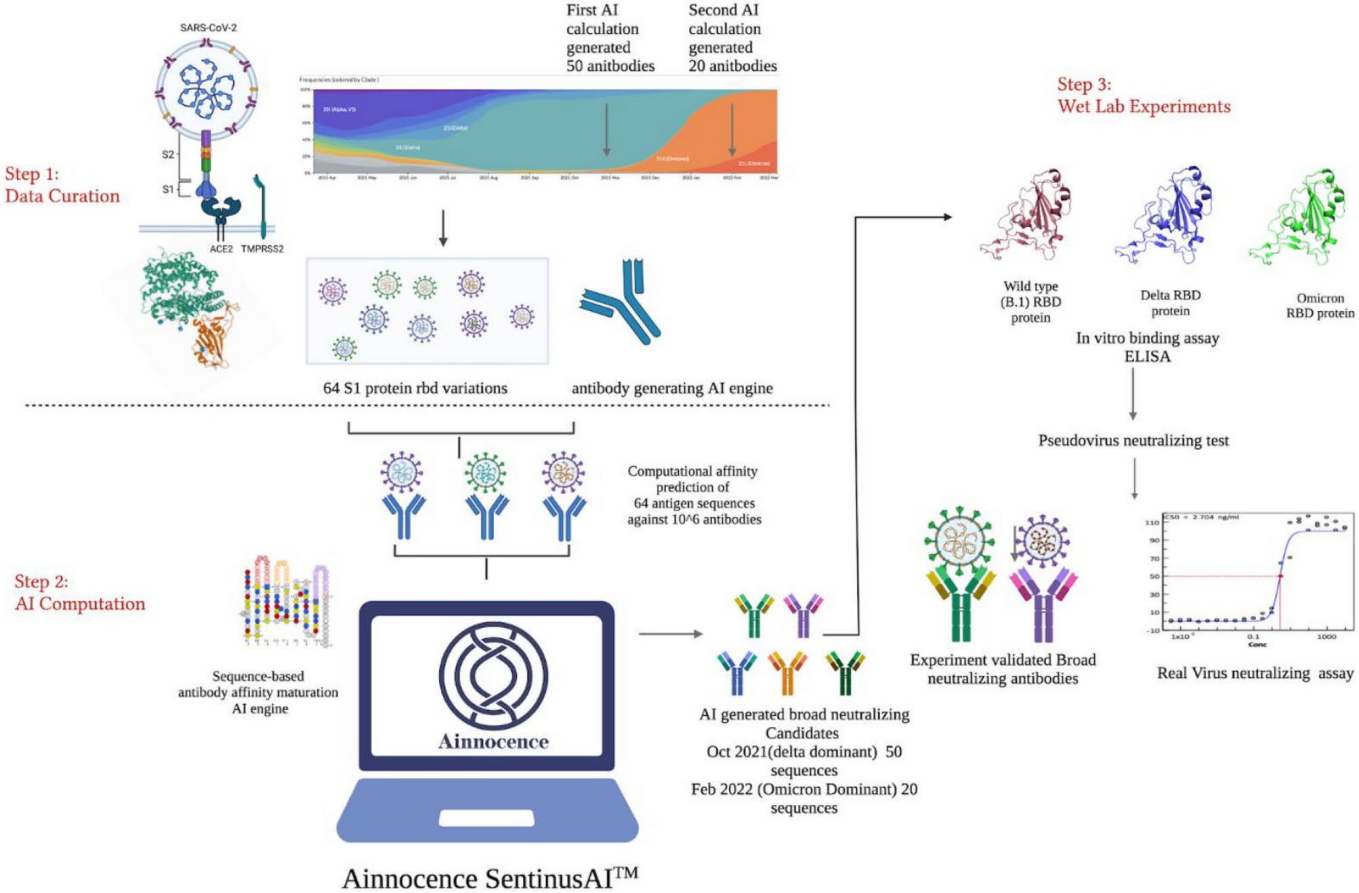
AI-based broad-neutralizing antibody design of SARS-COV-2.

Our in silico mutant library was constructed by considering only mutations on the template antibody paratope. The antibody paratope was mapped based on the crystal structure of the SARS-CoV-2 RBD in complex with template antibody CR3022 from the PDB bank [PDB ID: 6W41]. This is done only when a PDB structure is known and used. Otherwise, the antibody paratope is mapped using algorithms, such as ANARCI, that identify CDR1/2/3 regions in the heavy and light chains^30^. Interface contacts between antibody and antigen were identified within a 5.5-Å cutoff distance between the two amino acids’ side chain centers. We then generated single- and double-site mutations exhaustively within the paratope on both heavy and light chains to form an antibody somatic mutation library (Fig. 2: Step 1). This strategy generated more than 109 mutants from each antibody template (CR3022, Regen 10933, and Regen 10987).

Step 1: SARS-CoV-2 cross-binding sequence selection and virus mutation data curation. Step 2: AI-based antibody binding prediction and cross-variants binding selection for potential candidate sequences for future variants. Step 3: Measurement of antibody’s binding ability using an ELISA-based assay; and measurement of antibody’s neutralizing capacity using neutralization and cytopathic effect (CPE) reduction assays.

#### In silico library generation

The goal of the first round of in silico antibody design was to discover antibodies with improved binding affinities to over 1300 different historical SARS-CoV-2 strains, including the wildtype (B.1) and Delta strains. Therefore, antibody sequences were selected based on high cross-binding scores utilizing the trained machine learning model. These affinity scores (between each mutant antibody in the mutagenesis library and each unique mutant S1 protein of the historical SARS-CoV-2 strains) were computed for their VH and VL chains with the viral S1 protein sequences as the antigen. This score represents the likelihood of affinity improvement for a given antibody towards the targeting antigen. A score is generated for every antibody-antigen pair. For each SARS-CoV-2 spike protein mutant, the top 200 highest-scoring antibodies were selected from the 109 somatic mutation space as “strong binders” for that specific strain. All “strong binders” were then computed and selected following the above protocol for all 1300 SARS-CoV-2 strains (including B. 1 and Delta) as described in Step 2 of Fig. 2 where the 64 different antigen sequences were scored for all the antibody sequences in this library, and the final score is an average of the different antigen binding affinity scores.

SARS-CoV-2 variants evolve and acquire mutations in different regions of the S1 protein. Therefore, we hypothesized that antibodies that can bind to all observed S1 proteins may be able to bind to future S1 variants. From the common strong-binding antibody candidates for all 1300 variants. From these, we selected the top 50 cross-binding candidates with the highest average predicted score among all variants. This step concluded our first round of computations for cross-binding antibodies prior to Omicron.

We performed a second round of computation in February 2022 to further improve the Ab affinity towards Omicron. The same procedures detailed above were followed and the top 20 cross-binding antibody sequences with the highest average prediction scores were selected. This concludes our first-round computations for cross-binding antibodies prior to Omicron. This round of computation improved Ab affinity towards Omicron. The same procedures detailed above were followed and the top 20 cross-binding antibody sequences with the highest average prediction scores were selected.

### Wet lab experimentation

HEK293 antibody production, ELISA binding tests were conducted at Sino Biological Inc (1400 Liberty Ridge Drive, Suite 101, Wayne, PA 19087 USA). Coronavirus cytotoxicity assays were conducted by the Southern Research Institute (2000 9th Ave S, Birmingham, AL 35294).

#### ELISA

We measured each antibody’s ability to bind to the RBD of the different SARS-CoV-2 strains (B.1, Delta, Omicron) using ELISA. The ELISA used a coating buffer (500 mL) containing $\text{CBS}\left(0.75 \, \text{g}\right.$) and ${\text{NaCO}}_{3}/{\text{NaHCO}}_{3}(1.46$ g) at a $\text{pHof }9.6$. Each of the RBD proteins of the B.1, Delta, and Omicron strains were prepared at concentrations of $0.03$ and $1 \mu \text{g}/\text{ml}$, and then $100 \mu \text{l}$ of antibodies was added to each well. The plates were coated at $4{ }^{\circ }\text{C}$ overnight. After coating, the solution was removed by shaking and panting. The wells were then sealed with $2\text{\%}$ of BSA. The proteins were incubated for 1 h at room temperature. The solution was then discarded, and the proteins were washed twice with $300 \mu \text{l}$ of elution buffer (PBS buffer containing 2% Tween-$200,\text{pH }$7.2–7.4) and patted dry.

Each antibody was diluted to $1 \mu \text{g}/\text{ml}$ with the elution buffer containing $0.1\text{\% BSA}$. $\text{One hundred microx of}$ diluted antibody was then added to each well previously coated with RBD proteins. The solution was mixed evenly and incubated at room temperature for 2 h. The solution was discarded, and the antibody was washed three times with $300 \mu$ of elution buffer and patted dry. $\text{One hundred }\mu$ of Jackson: Goat Anti-Human IgG (H + L)/HRP secondary antibody was added to each well, mixed evenly, and incubated at room temperature for 1 h. The solution was then discarded, and the antibody was washed with $300 \mu$ of elution buffer and patted dry. The TMB substrate solutions $A$ and $B$ were mixed evenly at a ratio of 1:1, and $200 \mu$ of the mixture was added to each well and incubated at room temperature in a dark room. The reaction was stopped by adding $50 \mu$ of 2 M sulfuric acid to each well, and then the absorbance in each well was measured immediately at 450 nm. Overall, we generated ELISA data from $0.03 1\text{ug}/\text{ml}$ of RBD (WT, Delta, Omicron) proteins and 7 serial dilutions of the generated monoclonal antibodies to generate the IC50 values.

#### Coronavirus cytotoxicity assay

Viruses replicate by hijacking cellular mechanisms, leading to cell death; however, antiviral drugs can block this effect. We used a CPE reduction assay to determine whether neutralizing antibodies improved cell viability. This assay was performed with vero E6 cells expressing the ACE2 receptor, which mediates viral infection.

Cells were cultured in a minimal essential medium (MEM) supplemented with $10\text{\%}$ heat-inactivated fetal bovine serum (HI FBS). CPE and toxicity assays used cells that were collected from MEM supplemented with $1\text{\%}$ penicillin streptomycin glutamine and $2\text{\% HI FBS}$ and then resuspended to 200,000 cells per ml. Twenty microliters of the cell suspension (approximately 4000 cells) was added to each well.

Neutralization of the virus was detected by mixing a fixed number of virus particles with serial dilutions of the antibody, followed by the CPE assay. We added $5 \mu$ of serum-diluted antibody along with $5 \mu$ l of virus containing 1000 TCID in each well of a 384 -well plate. The plate was incubated for 1 h at ${37}^{\circ }\text{C}$, then the CPE assay was initiated by adding $20 \mu$ of the cell suspension. Blank controls consisted of only cells, while virus controls contained no antibody. The plate was incubated at $\text{in }5\text{\%}$ CO_2_ and 90% humidity for 72 h. Thirty microliters of the Promega Cell Titer-Glo Luminescent Cell Viability Assay Kit was added to each well and then incubated at room temperature for 10 min. Luminescence was read using a Perkin Elmer Envision or BMG CLARIOstar microplate reader to measure cell viability. Raw data from each well was normalized to the inhibition rates of 100% (without antibody) and 0 (for blank controls) to calculate % inhibition of CPE using the following formula:

$$\text{Inhibition rate}(\text{\%})=100\times \frac{\text{ (Test Value }-\text{Avg virus test va}\text{lue only})}{(\text{Avg blank test value}-\text{Avg virus test value only})}$$

The CPE assay was conducted in a biosafety level-3 laboratory using plates that had been sealed with a clear cover prior to reading.

Antibody cytotoxicity was detected employing antibodies that had been serially diluted with the same medium used in the CPE assay. A mixture consisting of $20 \mu \text{l}$ cells and $10 \mu \text{l}$ antibodies was added to each well in a multi-well plate. Wells containing cells only served as blank controls, while wells with cells treated with benzethonium chloride ($100 \mu \text{M}$ final concentration) served as negative controls. Luminescence was read as described in the CPE assay.

## Results

### Al modeling benchmarks

We investigated two different protein sequence modeling approaches to improve the prediction accuracy of antibody maturation based solely on sequence inputs. These modeling approaches, as shown in Fig. 1, include the graph-based approach and the language-based approach. The metrics are calculated based on the the “leave-5-out” (L5O) approach where the five-fold scores are averaged. Performance on previously unknown samples has been one of the most common challenges for deep learning neural networks. Our modeling aimed to improve the model’s robustness under a scenario of performing antibody affinity predictions. We investigated several neural network modeling approaches to find an optimized model for antibody screening, as shown in Table 1. The performance of each approach was assessed by evaluating the prediction accuracy on previously unseen samples.

**Table 1:** Benchmark performance comparison for baseline five-folds out-of-distribution validation.

Benchmark performance comparison: AUC (classification)			
Methods	AUC (standard deviation)		
bASA	0.63 (0.59–0.66)	AUC values are listed with the 95 percent confidence intervals	
DFIRE	0.65 (0.62–0.69)		
dDFIRE	0.59 (0.56–0.63)		
FoldX	0.70 (0.67–0.73)		
Discovery Studio	0.73 (0.70–0.76)		
SentinusAI®_**GNN**	**0.82 (0.07)**	AUC values are listed with the standard deviation among five folds	
SentinusAI®_**Bi-LSTM**	**0.73 (0.02)**		
SentinusAI®_ESM2 **Transformer**	**0.70 (0.02)**		

Benchmark performance comparison: Pearson (linear correlation)			
Methods	Pearson(r)		
bASA	0.22		
DFIRE	0.31		
dDFIRE	0.19		
Rosetta	0.16		
STATIUM	0.32		
FoldX	0.34		
Discovery studio	0.45		
SentinusAI® **GNN**	**0.61(0.10)**		
SentinusAI® **Bi-LSTM**	**0.40(0.06)**		

GNN & language model comparison: Spearman (ranking correlation)			
SentinusAI® graph-based	SentinusAI® Bi-LSTM-based	SentinusAI® ProtBERT transformer-based	SentinusAI® ESM2 transformer-based
0.54 (0.11)	0.37 (0.07)	0.45 (0.04)	0.43 (0.03)

Performances of classification were assessed by ROC area under curve (AUC). The correlation between experimental affinity changes and predicted values was evaluated using the Pearson and Spearman correlation coefficients.

**Fig. 3:**
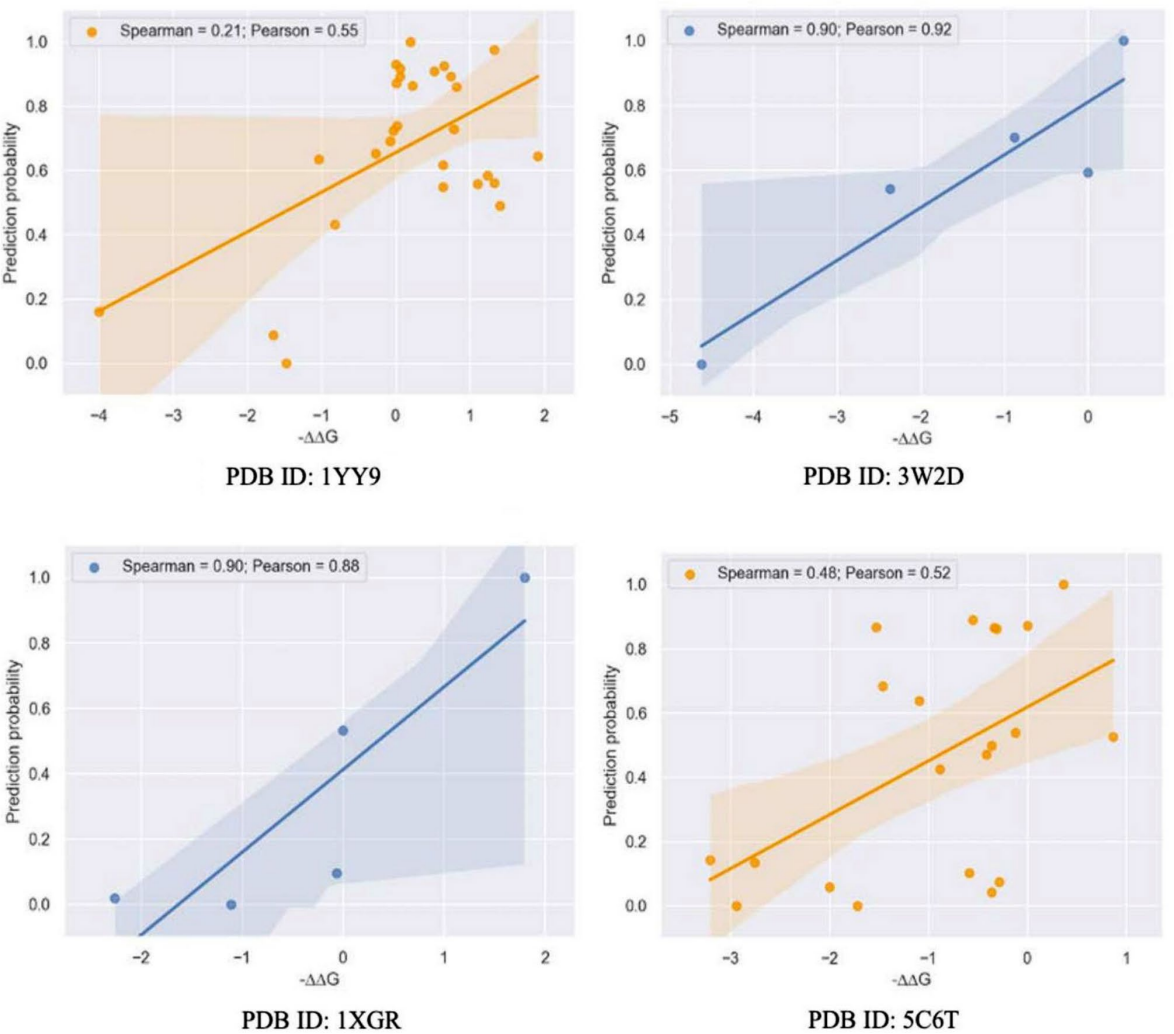
Scatterplot of model-predicted probabilities with experimental ΔΔG values for four representative complexes (wildtypes and mutations) using the out-of-distribution test set. Each of the targets in the independent test sets have a similarity score less than 0.21 relative to each other. The x-axis represents −ΔΔG value, which positively correlates with the affinity strength of each sample, while the Y-axis represents model-predicted probabilities. The correlation was quantified by Pearson’s correlation values and Spearman’s correlation values, respectively.

We also compared the ranking abilities of the different learning-based approaches, based on Spearman ranking coefficients (Table 1) where a tokenizer from the ProtBert or ESM^20^ transformer model was used to encode the sequences from our training data. The graph-based model (Spearman = 0.54) outperformed the language-based approaches, but it was observed that the Transformer-based models have powerful embeddings that can capture effective data from protein sequences at a nearly equal level. In addition, we evaluated the model’s prediction performance on several unknown complexes shown in (Fig. 3).

Table 1 shows the averaged AUC of binary classification on improved vs. weakened binders using language-based and graph-based models. Benchmark studies from^16^ are listed for comparison.

The performances of both the graph-based (AUC = 0.82) and language-based (AUC = 0.73) modeling approaches in distinguishing between strengthened and weakened binders were better than or comparable to that of Discovery Studio^16^, which is an often-used non-machine learning commercial structure-based approach. Unlike Discovery Studio, which employs a physical model derived from primary, secondary, and tertiary protein structure to compute binding affinity, our model learns the mapping between antibody sequence and binding affinity from a large amount of experimental data.

We then compared the model’s Pearson coefficient to those of prior works^16^ to examine the linear correlation between predicted values and experimental affinity changes due to mutations (Table 1). The graph-based model (Pearson = 0.6) outperformed most conventional (structure-based) in silico approaches, whereas language-based prediction yielded a Pearson coefficient of 0.40, which is comparable to that of Discovery Studio (Pearson = 0.45). These findings demonstrate that deep learning-based representations can be utilized to predict the binding of completely unknown variants during antibody maturation meaning that the model captures transferable features that contribute to the binding strength in antibody-antigen interactions.

In summary, both graph- and natural language-based approaches were able to predict interactions with novel variants during antibody maturation. We hypothesized that the model captures the key transferable features for predicting binding strength in antibody-antigen interactions.

### Affinity maturation efficiency

Our proposed modeling strategy significantly reduces complexity by allowing SentinusAl® to search large mutation spaces at a very low computational cost. Table 2 compares the efficiencies of in silico affinity maturation with those of two conventional structure-based approaches. Prodigy (PROtein binDIng enerGY prediction) is a collection of web services that provide predictions of binding affinity in biological complexes; and they also identify biological interfaces from crystallographic information^31^. Meanwhile, Schrodinger Desmond is a molecular dynamics tool used to calculate free energy values (i.e., binding affinity)^32^. Both approaches require complex structural input. Our approach uses an arbitrary antibody sequence template with a defined region of interest (ROI) of 20 amino acids. Single- and multi-site point mutations are performed within the ROI, followed by affinity computation (screening) of the resulting mutation space. Table 2 shows the computational time costs based on different sizes of virtual libraries using sequence-based and structure-based approaches.

**Table 2:** Computational cost of antibody maturation space search (sequence length $=20$ amino acids).

Methods	Input	Single-site mutations	Double-site mutations	Three-site mutations	Four-site mutations
	Mutation space	${10}^{2}$	${10}^{4}$	${10}^{6}$	${10}^{8}$
Schrodinger Desmond^32^	Structure	4 days	$\sim 100$ days	Not applicable	Not applicable
Prodigy^31^	Structure	336 s	17.7 h	89 days	Not applicable
SentinusAI®	Sequence	$<2$ min	6 min	40 min	3 days

*Not applicable: computational time $>100$ days.

### Broad coronavirus neutralization

**Fig. 4:**
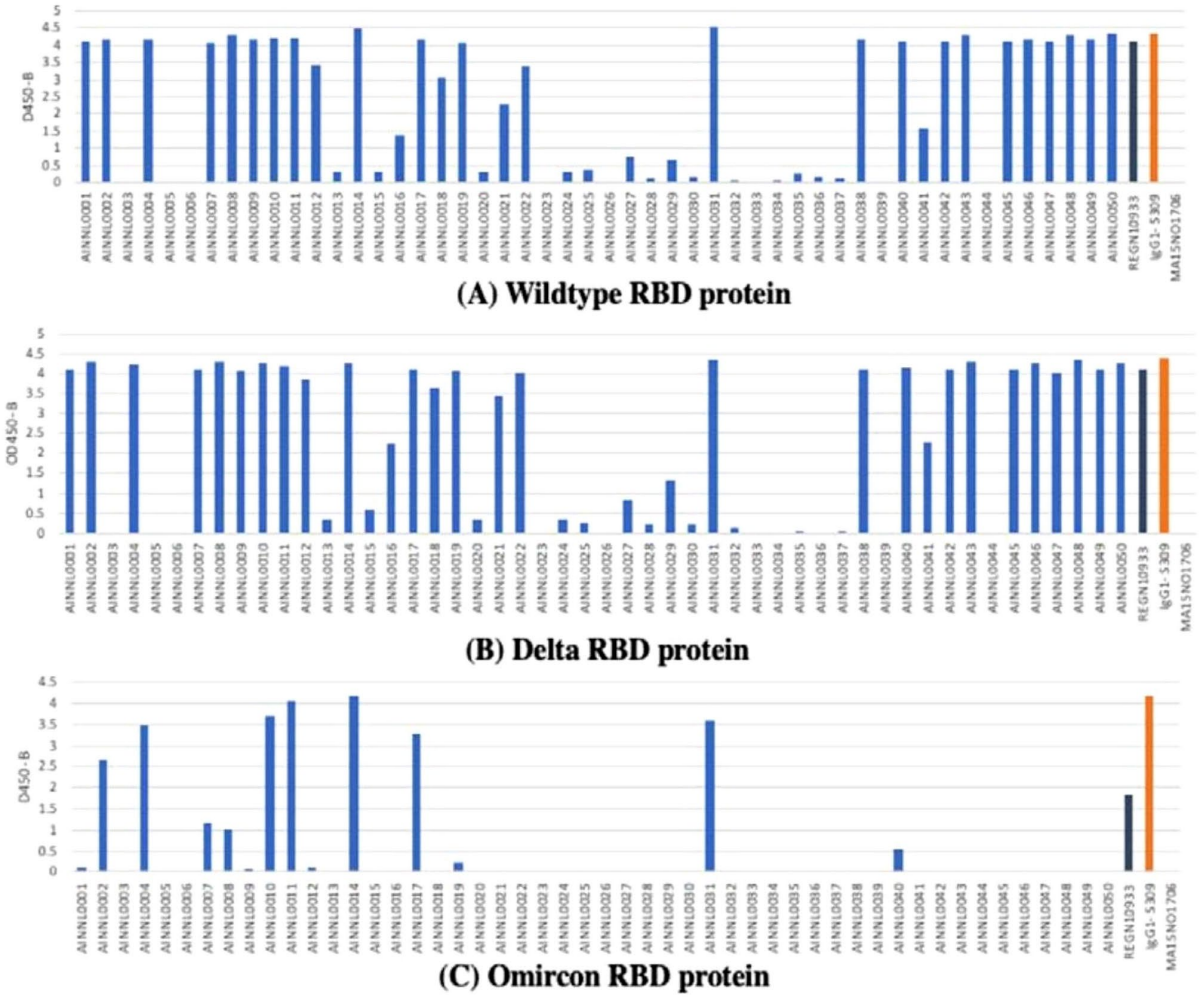
OD 450 absorbance values from direct ELISA of 50 antibodies ($1\mu \text{g}/\text{ml}$; blue) against wild type (**A**), Delta (**B**) and Omicron (**C**). The plate was coated with $1\text{ug}/\text{ml}$ RBD protein. OD450 $=$ optical density at 450 nm; two therapeutic SARS-CoV-2 antibodies were tested as controls (black, orange) for the variants.

We selected the top 50 AI-designed antibody sequences (AINL1-AINL50) with the best predicted binding abilities to the RBD. These sequences were synthesized and functionally assessed in the first round (pre-Omicron). Most of the antibodies synthesized were able to bind to the RBD of the SARS-CoV-2 spike protein, usually reaching an oversaturated state (i.e., OD value > 2.0) at the highest concentration tested. Some antibodies also bound well to the SARS-CoV-2 RBD protein at the lower concentration of $0.03\mu \text{g}/\text{ml}$ (Fig. 4). Both the first (AINL1-AINL50) and second (AINL51-AINL70) batches of 50 and 20 antibodies, respectively, were well-expressed and had a high hit rate for binding to B.1, Delta, and Omicron. The first and second batches yielded $14\text{\%}$ and $40\text{\%}$ triple cross-binding hit rates, respectively, ELISA results of OD450 Result for First Round of 50 antibodies (Table S1) and Second Round of 20 antibodies (Table S2) are available in the supporting information.

**Fig. 5:**
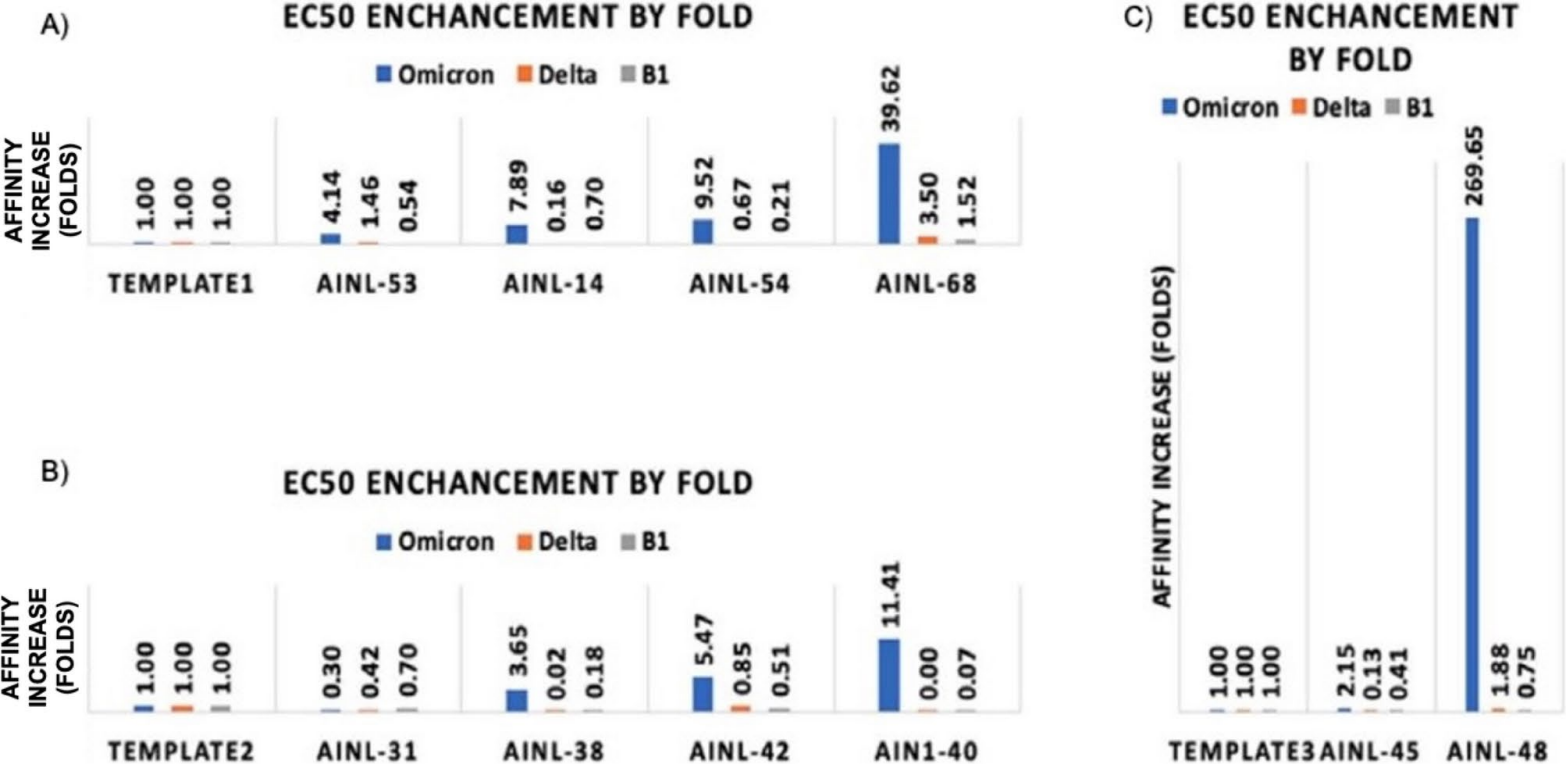
Affinity changes in designed antibodies compared to their template antibodies (**A**) Template 1 (CR3022) and associated AI-designed antibodies AINL53, AINL14, AINL54, and AINL 68. (**B**) Template 2 (Regen 10,933) and associated AI-designed antibodies AINL31, AINL38, AINL42, and AINL 40. (**C**) Template 3 (Regen 10,987) and associated AI-designed antibodies AINL45 and AINL48.

**Fig. 6:**
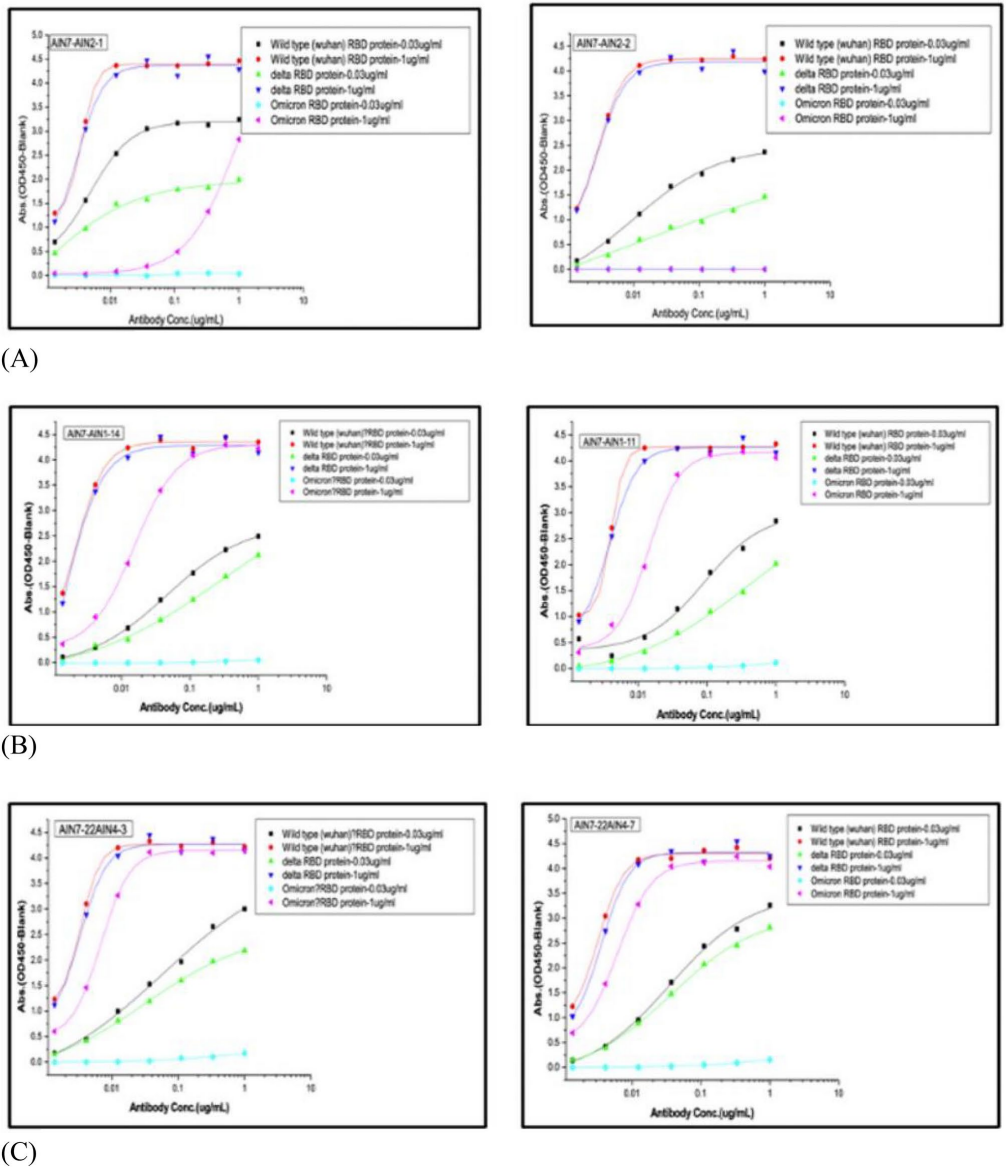
Selected ELISA curves for mAb binding to two different concentrations ($\text{and }1 \mu \text{g}/\text{ml}$) of the RBD of WT, Delta, and Omicron. (**A**) Controls of previously approved therapeutic antibodies; (**B**) first batch of AI-designed antibodies generated before Omicron prevalence; and (**C**) second batch of AI-designed antibodies generated after Omicron prevalence.

We then measured the fold-change in binding affinity improvement of the designed antibody versus the template antibody as an indicator of affinity maturation performance (Fig. 5). Qualitative differences in antibodies are reflected by the shape of dose-response curves for antibody binding (Fig. 6). The original data for the dose responsive curve IC50 (Table S3) for the positive hits are also available in the supporting information.

### Coronavirus cytopathic assay

**Fig. 7:**
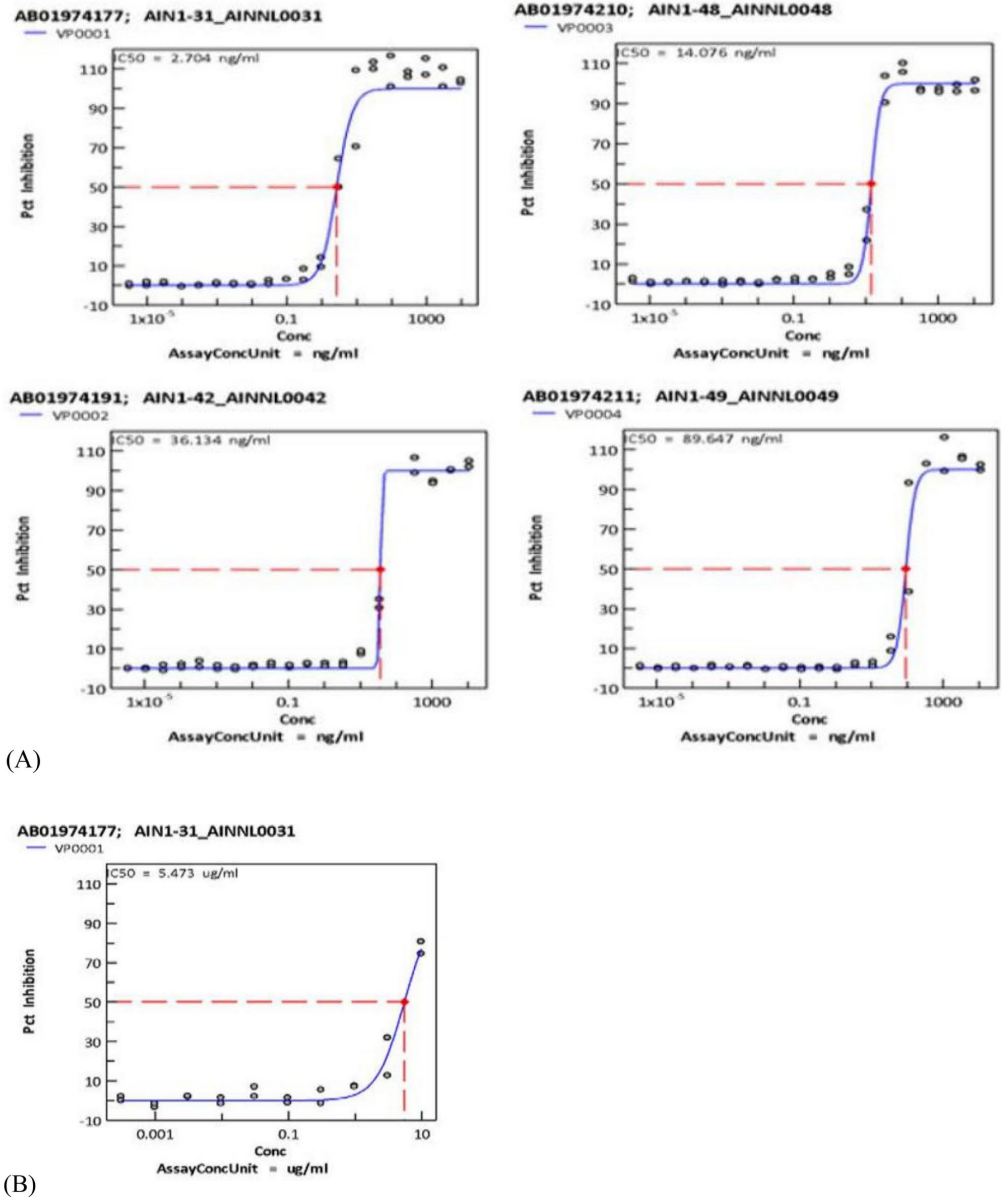
CPE inhibition curves of selected antibodies against delta (**A**) and Omicron (**B**). The virus was neutralized by mixing a fixed number of infectious virus particles with serial dilutions of the antibody and then adding this mixture to Vera E6 cells. Luminescence was read using a Perkin Elmer envision or BMG CLARIOstar plate reader after a 10 min incubation at room temperature to measure cell viability.

We next measured the ability of the designed antibodies to reduce the of the Delta and Omicron strains infecting Vero E6 host cells (Fig. 7). Ten antibodies neutralized the CPE of the Delta Strain at IC50 values of $<10\text{ug}/\text{ml}$, and one antibody neutralized the CPE of Omicron (Fig. 7 and Table 3). For example, the IC50 of AINNL0031 is $2.704\text{ ug}/\text{mL}$, indicating that it strongly neutralizes the CPE. Overall, none of the antibodies were significantly directly toxic with > 10uM CC50 against Vero E6 host cells. The anti-viral assay and cytotoxicity assay results of the first round of 50 antibodies (Table S4) against Delta and all 70 antibodies (Table S5) against Omicron are available in supporting information.

**Table 3:** Coronavirus cytotoxicity assay results on Delta*.

Sample ID	Main screen, IC50 (ng/ml)	Main screen, activity status	Main screen, max% inhibition
REGN10933	1.21	Active	159.93
AIN1-31_AINNL0031	2.704	Active	116.48
AIN1-48_AINNL0048	14.076	Active	109.96
AIN1-42_AINNL0042	36.134	Active	131.78
AIN1-45_AINNL0045	42.743	Active	102.12
AIN1-49_AINNL0049	89.647	Active	169.39
AIN1-50_AINNL0050	89.943	Active	108.57
AIN1-43_AINNL0043	196.18	Active	123.23
AIN1-38_AINNL0038	199.241	Active	160.54
AIN1-40_AINNL0040	1142.851	Active	138.69
AIN1-47_AINNL0047	1880.585	Active	117.5

*For more details, please refer to supporting information Tables S4 and S5.

## Discussion

Here, we tested whether of an Al-based, structure-free approach can design antibodies that are effective in vitro and at drastically reduced time and cost compared to those designed via traditional antibody engineering and affinity maturation strategies. This structure-free approach is critical, because although a large number of proteins can be sequenced using current sequencing technologies, determining their crystal structures remains a complex, risky, and time-consuming task. Here, we employed an in-silico antibody discovery approach that uses affinity prediction models and deep learning techniques to design antibodies from complex sequence information alone (i.e., without any structural information). This modeling strategy captures functionally critical features encoded at the amino acid level that contribute to the interactions and the resulting binding affinities between antibody and antigen. Modeling at the amino acid rather than the atomic level significantly reduces the time needed for both training and prediction; moreover, it can also accommodate smaller annotated training datasets due to the model’s simplicity.

The computational workflow efficiency increase is because the trained model does not use any structural information from the protein sequences. Instead, only combinations of sequence strings are used as inputs for this model. Therefore, this allows the AI model to be trained on sequence text strings which are one-dimensional instead of a three-dimensional structure PDB file, simplifying the computational complexity of training this model. One item to note from this approach is that some of the training data used in this model may have some implicit bias when selecting the paratope from the entire protein sequence as the training set from SKEMPI or AB-Bind are manually labelled from known proteins. However, it is also noted that when running prediction tasks, labelled fragments of a protein are also used when known, such as when known extracellular or secreted part of a protein are labelled in UniProt. This further simplifies the computational efficiency, but also does not consider the explicit structure, but only considers the labelled results as a method for further simplification of our computational process.

**Fig. 8:**
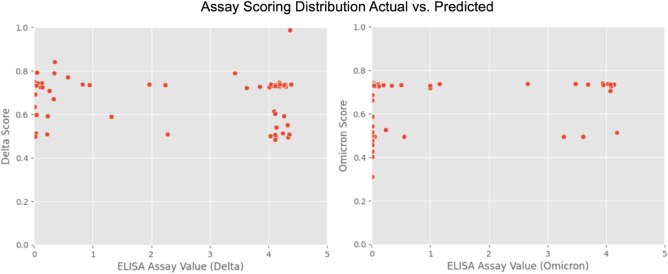
ELISA assay OD450 values compared to the model’s predicted score. Both delta and Omicron data are displayed in the scatterplots. The model has successfully predicted many of the ELISA-validated active antibodies (ELISA OD450 > 4) as the top-ranking model prediction scores (model score > 0.6).

The accuracy of our approach is higher than those of traditional methods; moreover, its reliability is also comparable to other methods featured in the benchmark study of traditional structure-based approaches on SKEMPI and AB-Bind datasets (Table 1). The results from the SARS-COV2 RBD work (Fig. 5) shows affinity improvements of 11.41-, 39.62-, and 269.65-fold compared to each original antibody template, even though the antigens were not part of the training set. These results demonstrate the model’s utility even for novel antigens. In addition, the resulting scores of the computation process is compared to the actual ELISA OD450 assay value shown in Fig. 8. In this figure, scatterplots are drawn for both Delta and Omicron antibodies. It can be seen that the top scoring AI antibodies are also positively correlated to the actual ELISA value, especially when comparing some of the most active antibodies confirmed using this assay (OD450 > 4). Although false positives are also found, this shows a much-improved overall hit rate compared to traditional methods such as using hybridoma clones. In a study for screening and selecting hybridomas producing antibodies targeting PD-1, only 51 out of 10,560 pools are identified as positive hits for their ELISA assay^33^. In terms of a secondary screening using flow cytometry, this workflow produced 5 hits from the primary 51 hits^33^. When directly comparing to our AI-based method, material costs and experimentation time with cell culture and hybridoma generation are directly saved as the AI workflow directly outputs monoclonal antibodies for expression and binding assays and resulting in an overall higher hit rate with significantly less samples for experimentation.

Interestingly, we found a discrepancy between the antibody binding data obtained through ELISA and the observed neutralizing capacity of the antibody for blocking infection. This difference may be due to a number of factors, including differences in RBD structure when the protein is plate-bound in the ELISA versus its structure when interacting with a live virus or in the presence of different antibody binding sites on the RBD (e.g., antibodies having high-affinity interactions that may not physically block the RBD and receptor interaction).

Our Al model was also able to design cross-binding antibodies against many different antigen populations, including viral mutant strains. The cross-binding antibodies may be capable of mutation-resistant binding to future evolving RBD region variations; therefore, the neutralization potency of such antibodies may be broad, which is a key characteristic of therapeutic antibodies targeting rapidly mutating viruses such as SARS-CoV-2. We hypothesized that the high dimensional features learned during the Al training process may represent components of the viral evolutionary process. This results in an ability to predict binding affinity in the virtual screening process even though the process considers only known virus strains. This approach was successful for SARS-COV-2, where $14\text{\%}$ of the 50 screened antibodies generated prior to Omicron were able to bind to all the strains (Omicron, Delta, and wild-type). Cross-reacting antibodies may also be less specific^34^; however, a broadly cross-reactive antibody (e.g., generated through engineering approaches^35^) may have better therapeutic potential in coping with viral evolution.

Our computational workflow allows for iterations with wet laboratory results to achieve better antibody design and cross-validation. This was demonstrated by the 40% cross-binding hit rate obtained in the second round of antibody synthesis (of 20 sequences), a considerable performance leap compared to the 14% cross-binding hit rate in the first round.

We have demonstrated a highly efficient and cost-effective approach for generating therapeutic antibodies for single or multiple viral strains. However, during the evaluation of this approach’s effectiveness, we found that the neutralizing capability of the designed antibody is not consistent with the binding affinity results. Aside from binding affinity, there are multiple mechanisms and processes involved in the neutralizing effects. For example, the binding epitope on the spike protein and antibody conformation also impact the infection and translocation process during viral invasion. Therefore, epitope mapping and conformation dynamics studies are required for a more precise design of neutralizing antibodies. In addition, we have not performed in vivo efficacy studies, which was beyond the scope of this research.

This study presents a novel approach with abilities to model the initial design of therapeutic antibodies and to iteratively improve upon these initial designs to account for future mutations in the target protein of a rapidly evolving pathogenic virus. Because our approach combines flexibility and high-throughput at a low computational cost, it can be beneficial in other applications of the technology as well.

## Supplementary Information

Supplementary Information.

## Data Availability

All data generated or analyzed during this study are included in this published article and its supplementary information files.
