# Supplementary material for: AI designed, mutation resistant broad neutralizing antibodies against multiple SARS-CoV-2 strains
Source: Sci Rep. 2025 May 3;15:15533. doi: 10.1038/s41598-025-98979-w (PMC12049519; doi:10.1038/s41598-025-98979-w)
Supplement: Supplementary file 1 — Supplementary Information. [file 41598_2025_98979_MOESM1_ESM.zip › SupportingInformation20250408/Figure S1.docx]

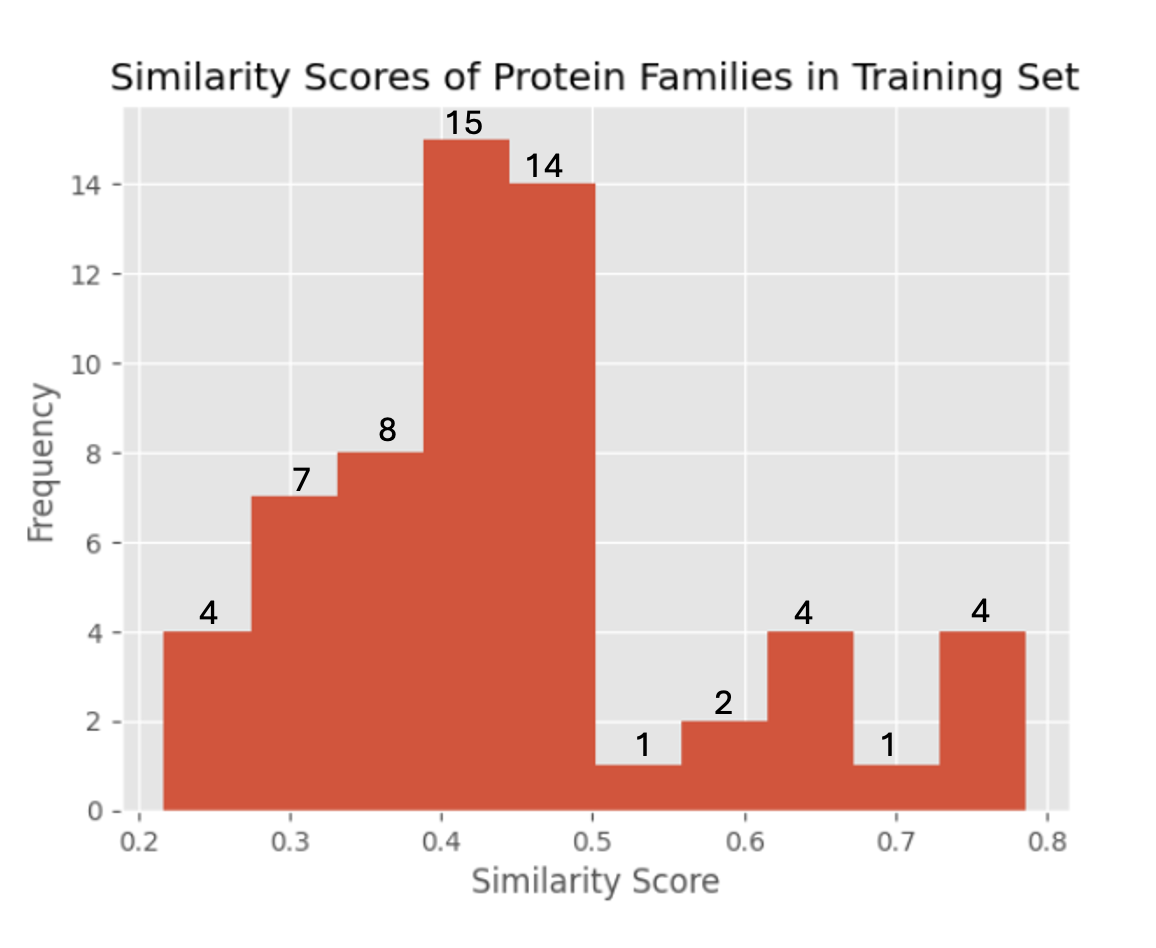


Figure S1: Similarity analysis of sequences in Leave-X-Out validation data compared to the other protein families in the training set. There are 60 protein families in the training set, and 5 are left out as validation data in each training fold. The remaining 55 protein families are used as training data. Overall, the similarity scores between the protein families in the training and left-out validation sets are low.
