# Supplementary material for: AI designed, mutation resistant broad neutralizing antibodies against multiple SARS-CoV-2 strains
Source: Sci Rep. 2025 May 3;15:15533. doi: 10.1038/s41598-025-98979-w (PMC12049519; doi:10.1038/s41598-025-98979-w)
Supplement: Supplementary file 1 — Supplementary Information. [file 41598_2025_98979_MOESM1_ESM.zip › SupportingInformation20250408/SupportingTables/SupportingTables Titles.docx]

Table S1: ELISA OD450 data for the first round of 50 Batch 1 antibodies along with reference controls. Binding to B.1, Delta, and Omicron were tested in this assay for this batch of antibodies.

Table S2: ELISA OD450 data for the second round of 20 Batch 2 antibodies along with reference controls. Binding to B.1, Delta, and Omicron were tested in this assay for this batch of antibodies.

Table S3: The original data for the IC50 dose response curve for both round 1 and round 2 batches of antibodies calculated from the ELISA assay data for binding to B.1, Delta, and Omicron

Table S4: Coronavirus cytopathic assay data report for the first round of 50 Batch 1 antibodies against Delta. This data is split into four sections:

1. Anti-viral assay compound dose-responsiveness data and activity status.
2. Cytotoxicity assay data and activity status.
3. Anti-viral assay and cytotoxicity assay results compared together.
4. Overall data report containing all of the data sections from above.

Table S5: Coronavirus cytopathic assay data report for the first round and second round antibodies for a total of 70 against Omicron. This data is split into four sections:

1. Anti-viral assay compound dose-responsiveness data and activity status.
2. Cytotoxicity assay data and activity status.
3. Anti-viral assay and cytotoxicity assay results compared together.
4. Overall data report containing all of the data sections from above.
